# Harm, benefit and costs associated with low-dose glucocorticoids added to the treatment strategies for rheumatoid arthritis in elderly patients (GLORIA trial): study protocol for a randomised controlled trial

**DOI:** 10.1186/s13063-017-2396-3

**Published:** 2018-01-25

**Authors:** Linda Hartman, Linda A. Rasch, Thomas Klausch, Hans W. J. Bijlsma, Robin Christensen, Yvo M. Smulders, Stuart H. Ralston, Frank Buttgereit, Maurizio Cutolo, Jose A. P. Da Silva, Daniela Opris, Jozef Rovenský, Szilvia Szamosi, Leonie M. Middelink, Willem F. Lems, Maarten Boers

**Affiliations:** 1 Amsterdam Rheumatology and Immunology Center ARC, VU University Medical Center, Amsterdam, The Netherlands; 2 Department of Epidemiology and Biostatistics, VU University Medical Center, Amsterdam, The Netherlands; 3 Department of Rheumatology & Clinical Immunology, University Medical Center Utrecht, Utrecht, The Netherlands; 4 Musculoskeletal Statistics Unit, The Parker Institute, Bispebjerg and Frederiksberg Hospital, Copenhagen, Denmark; 5 Department of Internal Medicine, VU University Medical Center, Amsterdam, The Netherlands; 6 University of Edinburgh, Edinburg, Scotland; 7 Department of Rheumatology and Clinical Immunology, Charité – University Medicine Berlin, Berlin, Germany; 8 Research Laboratory and Academic Division of Clinical Rheumatology, Department of Internal Medicine, University of Genoa, Genoa, Italy; 9 Reumatologia, Faculdade de Medicina e Hospitais da Universidade de Coimbra, Coimbra, Portugal; 10 Carol Davila University, Boecharest, Romania; 11 National Institute for Rheumatic Diseases, Piešťany, Slovakia; 12 Department of Rheumatology, Institute of Medicine, University of Debrecen Faculty of Medicine, Debrecen, Hungary; 13 Middelinc, Utrecht, The Netherlands

**Keywords:** Rheumatoid arthritis, Elderly, Prednisolone, Glucocorticoids, Safety, Harm, Benefit, Cost-effectiveness

## Abstract

**Background:**

Rheumatoid arthritis (RA) is a chronic inflammatory disease of the joints affecting 1% of the world population. It has major impact on patients through disability and associated comorbidities. Current treatment strategies have considerably improved the prognosis, but recent innovations (especially biologic drugs and the new class of so-called “JAK/STAT inhibitors”) have important safety issues and are very costly. Glucocorticoids (GCs) are highly effective in RA, and could reduce the need for expensive treatment with biologic agents. However, despite more than 65 years of clinical experience, there is a lack of studies large enough to adequately document the benefit/harm balance. The result is inappropriate treatment strategies, i.e. both under-use and over-use of GCs, and consequently suboptimal treatment of RA.

**Methods:**

The GLORIA study is a pragmatic multicentre, 2-year, randomised, double-blind, clinical trial to assess the safety and effectiveness of a daily dose of 5 mg prednisolone or matching placebo added to standard of care in elderly patients with RA. Eligible participants are diagnosed with RA, have inadequate disease control (disease activity score, DAS28 ≥ 2.6), and are ≥ 65 years. The primary outcome measures are the time-averaged mean value of the DAS28 and the occurrence of serious adverse events or adverse events of special interest. During the trial, change in antirheumatic therapy is permitted as clinically indicated, except for GCs. Cost-effectiveness and cost-utility are secondary outcomes. The main challenge is the interpretation of the trial result with two primary endpoints and the pragmatic trial design that allows co-interventions. Another challenge is the definition of safety and the relative lack of power to detect differences between treatment groups. We have chosen to define safety as the number of patients experiencing at least one serious adverse event. We also specify a decision tree to guide our conclusion on the balance of benefit and harm, and our methodology to combat potential confounding caused by co-interventions.

**Discussion:**

Pragmatic trials minimise impact on daily practice and maximise clinical relevance of the results, but analysis and interpretation of the results is challenging. We expect that the results of this trial are of importance for all rheumatologists who treat elderly patients with RA.

**Trial registration:**

ClinicalTrials.gov, NCT02585258. Registered on 20 October 2015.

**Electronic supplementary material:**

The online version of this article (doi:10.1186/s13063-017-2396-3) contains supplementary material, which is available to authorized users.

## Background

Rheumatoid arthritis (RA) is a systemic, chronic inflammatory disease mainly characterised by cartilage and bone damage, progressive disability, decreasing quality of life (QoL) and premature death [1]. Both the disease and its treatment cause multiple comorbidities such as increased incidence of cardiovascular disease, diabetes mellitus, and osteoporotic fractures [1, 2]. Approximately 1% of the world population is affected by RA with a prevalence of 2% in people 60 years and older [3]. In Europe, the prevalence of RA is expected to increase along with the increase in the proportion of elderly in the population. Current treatment strategies have considerably improved the prognosis, but recent treatment innovations (especially biologic drugs and JAK/STAT inhibitors) have important safety issues and come at high societal cost [2, 4]; in addition, many patients still have smoldering progressive disease despite treatment [2]. Apart from treatment, societal costs are a result of functional disability, decreased societal participation, and reduced work capacity [1].

### Balance of benefit and harm of glucocorticoid treatment

Glucocorticoids (GC) exhibit unparalleled anti-inflammatory and immunosuppressive actions. The introduction of GCs in the 1950s was a revolution in the treatment of inflammatory diseases, including RA; it resulted in the only Nobel Prize awarded to a rheumatologist (Hench, together with Kendall and Reichstein in 1950). In those early years, enthusiasm generated by the initial results of unequalled efficacy in previously untreatable diseases led to the unrestricted use of high doses of GC. Unfortunately, such use disclosed a spectrum of adverse events that caused wide disillusionment and a still ongoing debate about the balance between the benefit and harm of GC treatment. Recent trials have suggested this balance can be favourable [5, 6]. In addition, a meta-analysis has proven beyond doubt that GCs at low doses slow the progression of joint damage in RA [7]. These findings have renewed and induced both European and US rheumatology associations to review their position, especially in early RA [8, 9]. Low-dose GCs (defined as a daily dose of 7.5 mg prednisolone equivalent or less) could prove an important co-treatment of both early and established RA in combination with other antirheumatic drugs. Apart from their clinical effects, low-dose GCs have the potential to reduce the need for expensive biologic agents. Unfortunately, clinical studies large enough to adequately document the long-term balance of benefit and harm of low-dose GCs are lacking. A limited set of high quality data from trials does not support strong claims of harm (as opposed to a wealth of observational studies with high potential for bias), but the generalizability of trial data is questioned [10]. The result is inappropriate treatment strategies associated with both under-use and over-use of GCs, and consequently suboptimal treatment of chronic inflammation in RA [11].

### Adherence

Medication adherence (or compliance; generally defined as the extent to which patients take medications as prescribed) is often below 50% in patients with chronic diseases such as RA [12]. The measurement of medication adherence in clinical trials is challenging, and many different methods have been tried [13]. Computerised devices or electronic monitoring devices assess adherence more accurately than self-reporting and prescription refill records. Linking electronic monitoring devices to apps on smart devices may improve adherence.

### The trial

This report describes the design of the Glucocorticoid Low-dose Outcome in Rheumatoid Arthritis (GLORIA) study, which compares the balance of benefit and harm of the addition of 5 mg/day prednisolone or placebo to standard of care for 2 years, in patients 65 years of age and older. A secondary objective is to study adherence and to test the effectiveness of smart device technology to improve adherence in a randomised sub-study. The report focuses on the occurrence and influence of co-treatment as a result of the pragmatic design and the interpretation of the outcomes of benefit and harm. The details of the sub-study about adherence are described in Additional file 1. The items addressed in this protocol are described in the standard protocol items: recommendation for interventional trials (SPIRIT) checklist in Additional file 2.

### Cost-effectiveness

Another aim of this trial is to study the cost-effectiveness and cost-utility of adding low-dose glucocorticoid to the treatment of patients with RA. The costs of current RA treatment are very high with mean costs estimated at €3000/patient/year, and €15.0000/patient/year for patients on biologic treatment [14]. Significant savings can be realised by improving the RA treatment strategy [14]. It is very likely that the optimised use of GCs in RA would allow for important savings, specifically by delaying or avoiding the need for expensive biologic drugs and other agents such as JAK/STAT inhibitors.

## Methods

### Study design

The GLORIA study is a randomised, double-blind, placebo-controlled pragmatic multicentre clinical trial (phase IV). Eight hundred patients with RA are planned to be recruited from rheumatology clinics in seven European countries: Germany, Hungary, Italy, the Netherlands, Portugal, Romania, and Slovakia.

Patients will be randomised to either prednisolone or matching placebo in a 1:1 ratio. Randomisation is stratified for participating study site, for prior exposure to GC, and for concurrent start or change of any other second-line antirheumatic therapy. The randomisation is integrated in an interactive web response system (IWRS) as part of the electronic data management system. The minimisation randomisation method [15] will be used. Minimisation is a method of randomisation that allocates subjects to the treatment group that best maintains balance in stratifying factors. It is effective even at small sample sizes and with multiple stratification variables [16].

### Eligibility criteria

The eligibility criteria are shown in Table 1.

**Table 1:** Inclusion and exclusion criteria for participating in the GLORIA trial

Inclusion criteria
1. Diagnosed with RA according to the 1987 or 2010 classification criteria of the American College of Rheumatology (ACR) and the European League Against Rheumatism (EULAR) [17, 18]
2. Inadequate disease control, as evidenced by a disease activity score of 28 joints (DAS28) ≥ 2.6, calculated with erythrocyte sedimentation rate
3. Age ≥ 65 years
Exclusion criteria
1. Having low probability of benefit
a. Change, stop or start of antirheumatic treatment in the last month prior to eligibility assessment, including methotrexate, sulfasalazine, hydroxychloroquine, leflunomide, azathioprine, intramuscular and oral gold, cyclosporine, biologic agents including anti-tumour necrosis factor (TNF), anakinra, abatacept, rituximab, tocilizumab
b. Treatment with systemic glucocorticoid (GC): oral or parenteral GC with a cumulative prednisolone equivalent dose of 200 mg or higher in the last 3 months.
c. Treatment with any GC (oral, intra-articular, intravenous or intramuscular) in the last 30 days
2. Having high probability of harm
d. Exposure to investigational therapy in the last 3 months
e. Current participation in another clinical trial
f. Major surgery, donation, or loss of approximately 500 ml blood within 4 weeks prior to the screening visit
g. Absolute contraindication to low-dose prednisolone, as determined by the treating physician, such as: uncontrolled chronic infections, diabetes mellitus, hypertension, osteoporosis. When these conditions are under control (e.g. with anti-osteoporosis drugs, antihypertensive drugs) these patients can enter
h. Absolute contraindication to calcium and/or vitamin D supplement as determined by the treating physician, such as hyperparathyroidism (when insufficiently treated)
i. Uncontrolled comorbidities, short life span, etc. as determined by the treating physician
3. Difficulty in measuring benefit/harm
j. Absolute indication to start with oral or intravenous GC, according to the treating physician
k. Inability to comply with medical instructions or inability to assess major outcomes
4. Not capable or willing to provide informed consent
Most exclusion criteria are temporary

Inclusion criteria: patients diagnosed with RA (1987 [17] or 2010 criteria [18]), inadequate disease control (disease activity score in 28 joints (DAS28) ≥ 2.6) [19], and age 65 years or older.

Exclusion criteria: these criteria are categorised as having low probability of benefit, having high probability of harm, difficulty in measuring benefit and/or harm, and patients not capable of or willing to provide informed consent.

### Intervention

Patients will receive either prednisolone 5 mg/day or matching placebo added to existing antirheumatic treatment, for 2 years. Subsequently, study medication is slowly tapered in linear fashion to zero over 3 months by inserting increasing numbers of non-treatment days every 2 weeks (first 2 weeks, no capsule on Mondays; second 2 weeks, no capsule on Monday and Thursdays, etc.). As in standard of care, all patients will receive daily calcium 500 mg and vitamin D3 800 IU. Dietary or life-style requirements will not be imposed upon patients because this is not done in standard care.

Patients may suffer from partial or complete unresponsiveness of the hypothalamic-pituitary-adrenal (HPA) axis in times of stress. Therefore, the following procedure is followed to prevent medical complications. No action is required for patients with minimal stress. Patients with mild stress will take one extra capsule of study medication for 1–2 days. Patients with moderate stress (i.e. surgical procedures, infections requiring parenteral antibiotics) will receive 25 mg hydrocortisone directly before the procedure and 8-hourly thereafter for 1–2 days or until recovery. Study medication can be restarted when the patient is back on oral feeding. For patients with severe stress, the same procedure as for patients with moderate stress is followed, except that patients with severe stress will be treated with 50 mg hydrocortisone. Patients do not need to be unblinded to treatment allocation if not required. They can restart study medication after the stress period has ended.

#### Use of co-interventions for the treatment of RA

Apart from the study medication all other treatment, except oral and intravenous GCs, is allowed; both treatment for comorbidities and antirheumatic treatment are allowed at the discretion of the treating physician. This includes biologic and non-biologic disease-modifying antirheumatic drugs (DMARDs), non-steroidal anti-inflammatory drugs (NSAIDs), acetaminophen, and short-term GC for comorbidities (e.g. chronic obstructive pulmonary disease). Patients requiring short-term oral GC for comorbidities can also stay in the study; such patients will continue to use study medication and will follow the normal assessment schedule.

To approach an initial “clean comparison” between treatment arms it is advised not to start other antirheumatic therapy (DMARDs, biologic drugs, etc.) or give intramuscular (i.m.) or intra-articular (i.a.) GC injections in the first 3 months. However, it is allowed if clinically judged as unavoidable. In such cases, preferred administration is at baseline. Patients developing an absolute indication for oral or intravenous GC therapy for their RA will discontinue the study drug, but will be retained in follow up and handled separately in the analysis. Patients undergoing emergency or routine surgery can be unblinded if parenteral GC is needed perioperatively. In the case of elective surgery, study medication can be given perioperatively as oral GC for adrenal crisis prophylaxis if local guidelines permit, thus obviating the need for unblinding.

#### Adherence

To measure adherence, an adherence medication packaging solution that combines objective therapy compliance monitoring technology and special software is delivered. All patients will have an adherence monitoring device loaded into the cap of the drug bottle. Caps will be collected and sent back to the manufacturer to be read centrally. Adherence data on all patients will be registered in the study database and analysed at the end of the study. In a sub-study an intervention to improve adherence will be tested (see Additional file 1).

### Study medication

Prednisolone 5 mg is bought commercially, and tablets of equal weight are manufactured; both are over-encapsulated to create the blinded study medication. These processes are performed by Bluepharma – Indústria Farmacêutica, S.A., which is located in Coimbra, Portugal, under Good Manufacturing Practice guidelines. Subsequently, Bluepharma ships the medication in controlled batches to each clinical site pharmacy, where it is securely stored in the original package under controlled conditions.

The local pharmacy supplies the study medication to the patients. At each visit, patients return their medication bottle and remaining capsules; the procedure, including capsule count, is logged.

### Procedures

**Fig. 1:**
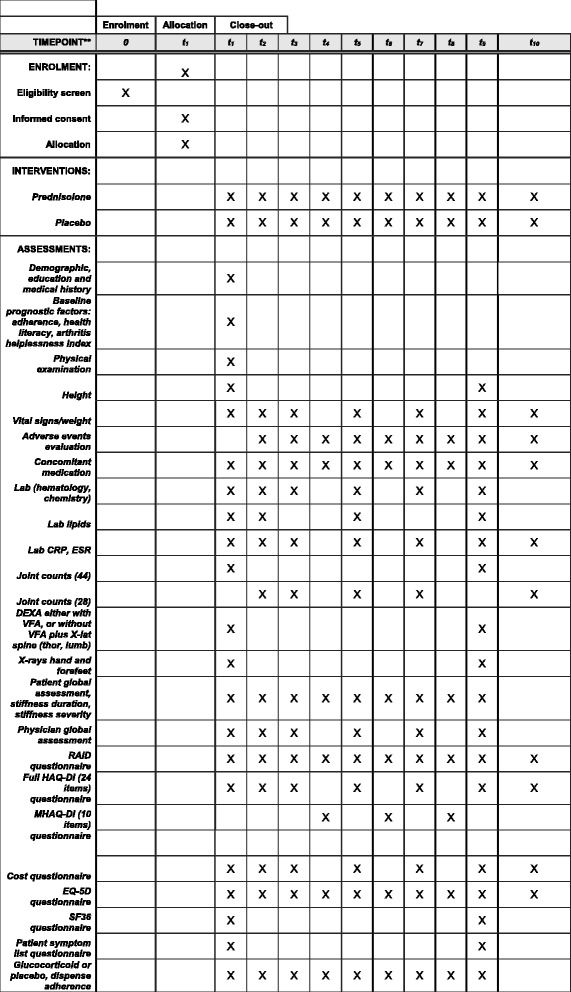
SPIRIT figure

Assessments comprise seven clinic visits and three assessments by telephone. Assessments include the collection of demographic and clinical data, through physical examination and questionnaires (Fig. 1). Almost all assessments (and their frequency) are routine and can be regarded as standard of care. The assessments are represented in the SPIRIT table in Fig. 1.

### Outcomes

Outcomes and variables will be collected at baseline and 3-monthly thereafter. After 2 years, trial medication will be tapered and stopped over 3 months.

#### Primary outcome measures

##### Benefit

Benefit will be measured on disease activity, i.e. time-averaged mean value of the Disease Activity Score of 28 joints (DAS28) and on progression of joint damage. The DAS28 is an index calculated from counts of painful and swollen joints (28 joints, i.e. shoulders, elbows, wrists, metacarpophalangeal and proximal interphalangeal joints, and knees), erythrocyte sedimentation rate and patient global assessment [19]. The DAS28 will be performed at all clinic visits, except for the baseline and the 2-year visit. During these two visits a more comprehensive DAS of 44 joints (DAS44) will be performed [19]. Damage progression is measured by 2-year change in total Sharp/van der Heijde damage score assessed on radiographs of the hands and forefeet [20].

##### Harm

Contrary to most drug trials, GLORIA designates harm as a co-primary outcome because the intake of prednisolone is driven mostly by perceptions of harm. Assessing harm in trials is challenging because the incidence of serious adverse events (SAEs) is usually rare, leading to power issues [21]. As clinical concern about the use of GCs relates to many different events, we decided to create a composite definition of harm, comprising the number of patients with at least one SAE or an adverse event (AE) deemed of special interest (defined below). We expect the proportion of patients meeting this definition to be about 20% in the control group, which allows detection of meaningful differences with the GC group at the chosen sample size.

Harm will be evaluated by spontaneous descriptions of the patient or physician, and by the results of a 53-item symptom list completed by the patients at the beginning and end of the study. AEs deemed of special interest are defined as follows:

- Any AE (except loss of efficacy, worsening of disease) that leads to the definite cessation of one of the antirheumatic drugs, including trial medication
- Cardiovascular event (myocardial infarction, cerebrovascular event, peripheral arterial vascular event)
- Newly occurring hypertension requiring drug treatment
- Newly occurring diabetes mellitus requiring drug treatment
- Symptomatic bone fracture requiring treatment
- Infection requiring antibiotic treatment
- Newly occurring cataract or glaucoma

Where appropriate, both incidence rates of SAEs and AEs of special interest and sufficient detail on the clinical characteristics of SAEs and AES, such as severity, frequency, and timing, will be reported [22]. AEs of special interest will be adjudicated by requiring a declaration from the site physician, backed up by source documentation on site (e.g. correspondence on hospital admission).

#### Secondary outcome measures

Demographic measurements, medical history, and prognostic factors (including risk factors for glaucoma, education, adherence, health literacy and arthritis helplessness index) are assessed at baseline.

Cost data will be collected by a cost-questionnaire completed 3-monthly. For cost items, unit prices calculated in previous studies and tariffs will be used. Costs that will be estimated are direct health care costs (i.e. costs of treatment and monitoring, including prevention and treatment of side effects) and indirect costs (based on activity limitations valued at shadow price for patients not working, and work disability for those still holding a paid job). Production losses will be valued using the friction cost method: only sick leave during a friction period (23 weeks) needed to replace a person is taken into account.

Secondary outcome measures regarding benefit and harm of prednisolone or placebo include the World Health Organization-International League of Associations for Rheumatology (WHO-ILAR) core set of RA outcome measures [23], morning stiffness, fatigue, bone mineral density and vertebral fractures. For a full listing see Additional file 3.

### Medical emergencies

Each randomised subject will be provided with a card detailing emergency contact details. Subjects will be requested to carry this card with them at all times whilst participating in the trial. In the case of emergency and if clinically necessary, the study team can contact the unblinded reviewer to break the code.

The Sponsor has clinical trial insurance, which is in accordance with relevant legislation in the participating countries. A patient will receive appropriate compensation in the case of any injury caused by participation in the trial. The insurance applies to damage that becomes apparent during the study or within 4 years after the end of the study.

### Data management and monitoring

During the study, clinical trial management and monitoring is performed by a clinical research organisation. The information required by the protocol is entered into an electronic data collection system. Plausibility checks will be performed according to a data validation plan. After all data are entered and all queries are resolved, the database will be closed.

### Sample size calculation

In the chosen analysis strategy, to detect differences in benefit (disease activity), extensive RA trial experience (both for GC and other agents) has shown a sample size of 200 patients per group is amply sufficient [5]. For harm, the true incidence of AEs with GC treatment is currently unknown. Most relevant data to assess sample size adequacy for this study come from the CAMERA-2 trial [6].

Based on a one-sided *Z* test, at a base case expectation for a total of 20% of patients experiencing at least one event in 2 years in the placebo group and 400 patients in each treatment group, we have about 80% power to detect an increase of 7% (from 20 to 27% events; 90% power for an increase of 9%). These estimates change little when the base case expectation is varied by ± 5%.

At the chosen sample size, and observed placebo event rates between 15 and 25%, observed point estimates of difference of 4–5%, respectively, will have a one-sided *p* value < 0.05 and thus be declared significant. In case the trial detects smaller, non-significant differences in AEs favouring placebo, the upper 95% confidence bound can be calculated to be about 3–4% above the point estimate. For example, if the trial shows a non-significant difference of 3% more AEs in the GC group, this finding is compatible with a real increase not exceeding 6%.

### Statistical analyses

#### Hypotheses

We will test hypotheses about the differences in benefit (DAS28 score and damage progression) and harm (encountering an adverse event, as defined in the protocol) of prednisolone treatment versus placebo. We state two sets of one-sided null hypotheses about treatment effects of prednisolone, one set for benefit, the other for harm. The hypotheses and their tests are one-sided in view of pre-existing knowledge on benefit and harm.

Under the null hypothesis, we expect to find no difference in decrease in DAS28 and in joint damage progression between the prednisolone and the placebo group after 2 years (primary benefit null hypothesis); and after 3 months (secondary benefit null hypothesis for DAS28). Second, under the null hypothesis we expect to find no difference in selected AEs (as defined in the protocol) between the prednisolone and placebo group after 2 years (primary harm null hypothesis) and 3 months (secondary harm null hypothesis).

#### Harm and benefit analyses

We will estimate the average effect of treatment on continuous outcomes (e.g. DAS28 and on damage progression) in separate mixed-effects regression models [24]. We will apply a logistic mixed-effects regression model to estimate the probabilities of harm and to test the difference in odds. In all models we account for the stratification of the GLORIA design.

Quantifying the power of tests in mixed-effects models is difficult as it is based on untestable assumptions. However, we expect that the mixed-effects models will increase power over a simple *Z* test, discussed in the previous section, due to the longitudinal structure of the data.

Primary analyses will be initially performed with the three stratification factors and the interaction with treatment allocation (country, prior GC exposure, change of antirheumatic treatment at baseline) in the model. Non-significant factors will be excluded from subsequent models.

##### Missing data

In the analysis with the first benefit outcome of disease activity, operationalised as DAS28, we first assume that missingness occurs at random [25]. The mixed-effect model will be estimated by full information maximum likelihood [25], which assumes that missingness can be explained by the observed data. In addition, for dropouts we investigate the possibility that missingness occurs not at random: we will do a worst-case analysis where we assume that patients with missing DAS28 data after a certain date until the end of the 2-year trial period did not respond or lost response (non-responder imputation). This imputation method recodes the DAS28 value of all those missing assessments to the patient’s baseline value.

The way of handling missing data for the second benefit outcome of damage progression, operationalised as difference between Sharp van der Heijde radiograph scores at baseline and at 24 months, depends on which value is missing. We will exclude patients with missing data at both time points or at the 2 year measurement list-wise from this analysis. In patients missing only a baseline value, this value can be imputed by linear interpolation from values of an older X-ray, if present; otherwise such patients will also be excluded. In the second step, we will extend the first analysis for a multiple imputation analysis of missing outcomes using the algorithm “multiple imputation by chained equations” (MICE) [26].

For the harm outcome, operationalised as the occurrence of at least one serious AE or an AE deemed of special interest, for most dropouts from the study we will have information to indicate the presence or absence of an AE at the last follow-up visit. If this information is not present, we will first create a dataset under the assumption that no such AE occurred in these cases (best case analysis); and then we will create a dataset under the assumption that such an AE did occur in these cases (worst case analysis).

##### Classification of co-interventions

Given that GLORIA is a pragmatic trial where co-interventions are allowed, the observed effects are likely a compound of the “pure” treatment effects and the effects of (potentially differential) co-interventions in the treatment and placebo groups. In theory, two types of confounding can be distinguished - harm and benefit confounding. Harm confounding does not play a role.

**Fig. 2:**
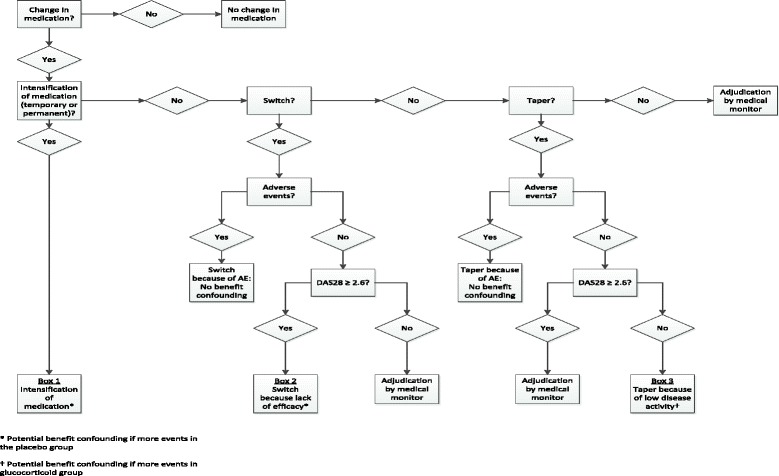
Decision tree for the assessment of benefit confounding in an individual patient

Benefit confounding can be defined as the expectation that patients on placebo more frequently require co-interventions because their RA is not under control, leading to subsequent improvement in disease activity. Benefit confounding will most likely work in one direction: it will decrease the difference in DAS28 or damage progression between the prednisolone group and the placebo group; so if the trial shows that the prednisolone group has significantly more DAS28 improvement and less damage progression (primary objective), benefit confounding no longer impacts the main conclusion of the trial. If the primary objective is not met we will proceed to assess the presence of benefit confounding. First, we will study co-medication changes and distinguish three types: (co-medication) intensification, switch and taper. This forms the basis of a decision tree (Fig. 2).

The first type, permanent or temporary intensification of co-medication, comprises an increased dose of current antirheumatic drug(s), the addition of a new antirheumatic drug to the current treatment, or an i.m. or i.a. GC injection (Fig. 2, Box 1). In the case of intensification, lack of efficacy can always be assumed.

The second type, co-medication switch, can be for lack of efficacy or for an AE, as documented by a high DAS28 or an AE report (Fig. 2, Box 2). The medical monitor will adjudicate situations where neither of these can be documented.

The third type, co-medication taper, comprises a decrease in dose or stop of current medication(s) without initiating other antirheumatic therapy. The main reason to taper is low disease activity, sometimes in the presence of an AE (Fig. 2, Box 3). The medical monitor will adjudicate situations where neither of these can be documented.

For practical reasons the analyses regarding benefit confounding are limited to the first “medication change event” in the case of multiple events in the same patient. If significantly more treatment intensifications, more switches for lack of efficacy or fewer tapers for low disease activity occur in the placebo group than in the prednisolone group, we will assume benefit confounding. As we assume that benefit confounding works in one direction, we will use one-sided log likelihood ratio tests for differences in the occurrences of the three types of co-medication changes (intensification, taper and switch) between the prednisolone and placebo group.

If there are no differences between the groups in co-medication changes we will proceed to the next step. This is to study the 3-month results in the subgroup of patients who started only study medication at baseline, i.e. who had no changes in co-medication at baseline or in the first 3 months. We will assume benefit confounding if two conditions are met: (1) prednisolone benefits these patients more than placebo, i.e. a significant difference in ∆DAS28 over 3 months, and (2) this improvement in disease activity is maintained in the remaining trial period, i.e. mean DAS28 in the prednisolone group does not deteriorate more than 0.6 between 3 and 24 months.

If these conditions are not met, we will proceed to the final step. We will compare the number of GC injections between the groups. If there are significantly more injections in the placebo group, we will assume benefit confounding. The presence of benefit confounding will be used in our interpretation of overall trial results.

##### Interpretation rules

**Fig. 3:**
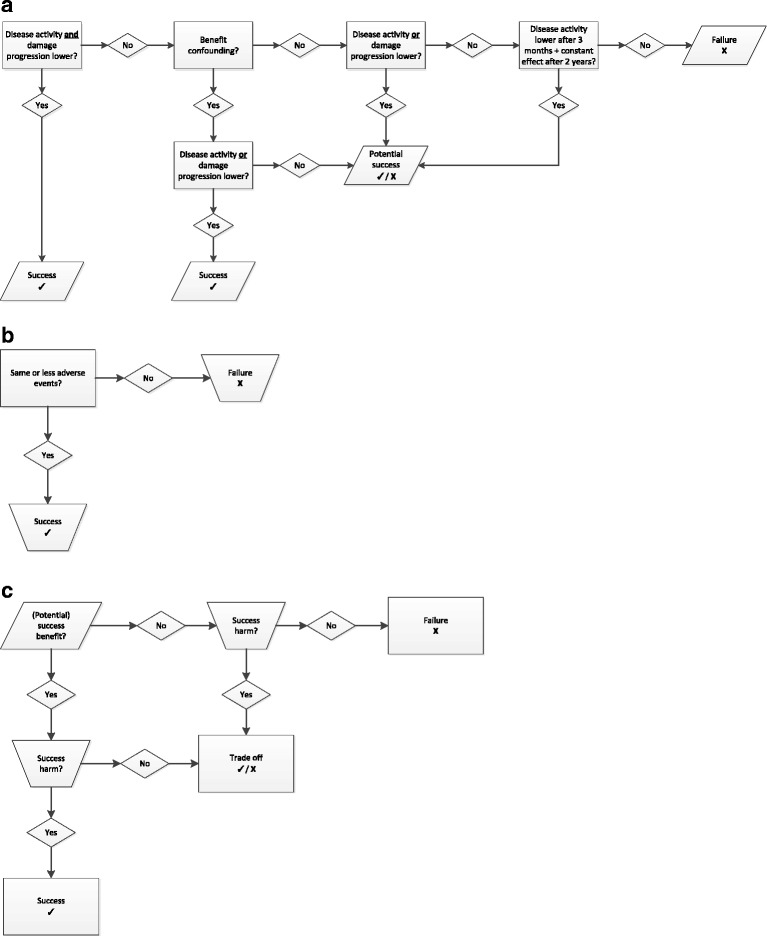
Interpretation rules for the assessment of the benefit and harm outcomes: prednisolone versus placebo group. **a** Interpretation rules for benefit: prednisolone versus placebo group. **b** Interpretation rules for harm: prednisolone versus placebo group. **c** Combined assessment for benefit and harm: prednisolone versus placebo group

The interpretation of the trial result with two primary endpoints is a challenge. In this trial we want to summarize the results in one outcome measure because both primary endpoints are equally important for the final assessment of the trial result. Therefore, we defined a composite outcome measure, which assesses the outcomes of benefit (disease activity and damage progression) and harm simultaneously. We will compare the prednisolone group to the placebo group and we will interpret the results as presented in Fig. 3.

For benefit, the trial is a success if the disease activity and damage progression are both lower in the prednisolone group (see Fig. 3a). In addition, it is a success if either disease activity or damage progression is lower in the prednisolone group, in the presence of benefit confounding. Finally, it is a potential success if only benefit confounding is demonstrated. For harm the trial is a success if the occurrence of AEs is the same or lower in the prednisolone compared to the placebo group (see Fig. 3b).

The assessment of benefit and harm are then combined for a final assessment (see Fig. 3c). Success in (potential) benefit and harm obviously translate into overall success, whilst other combinations result in a tradeoff or failure. We assess the final outcome as a failure if both benefit and harm do not result in success. The tradeoff assessment is defined as a failure in (potential) benefit, whilst harm was assessed as a success. The other possibility for tradeoff is a success in (potential) benefit and a failure in harm.

### Cost analyses

The analyses of cost will be driven by the primary analysis result and interpretation. If the combined assessment of benefit and harm is a success (both benefit and harm favour GC), cost-effectiveness analysis will be performed with the mean DAS28 as the effectiveness measure. If the combined assessment is a failure, a simple cost comparison will be performed. Finally, if the combined assessment points to a tradeoff, a cost-utility analysis will be performed. The reason is that there is no simple metric for effectiveness other than the utility to describe the potential benefit of treatment in this situation.

The cost analyses will be performed according to the intention-to-treat principle and from a societal perspective. Analysis will involve models of cost per unit of efficacy. Different outcome measures will be used as unit of efficacy. Confidence intervals (CI) for costs and differences in costs will be estimated using bias-corrected accelerated bootstrapping with 1000 iterations.

The incremental cost effectiveness ratio (ICER) will be calculated by dividing the difference between the mean costs by the difference in mean effects of the two treatment groups. CIs for the ICER are also calculated using bootstrapping with 5000 iterations. The results will be graphically presented on the cost-effectiveness plane and a cost-effectiveness acceptability curve will be constructed. Sensitivity analysis will be done to assess the effect of uncertainty in the main cost drivers and utility estimates on the ICER.

### (Serious) adverse events

Subjects will be carefully monitored throughout the study for SAEs and suspected unexpected serious adverse reactions (SUSARs). All SAEs and SUSARs will be reported by the investigator to a pharmacovigilance manager within 24 hours or 7 days of being aware of them, respectively.

All SAEs and SUSARs that are possibly, probably or definitely related to the investigational medical product are subject to expedited reporting to regulatory authorities and ethics committees, in accordance with the International Council on Harmonisation of Technical Requirements for Registration of Pharmaceuticals for Human Use (ICH) guidelines for Good Clinical Practice (GCP) and the EU Directive 2001/20/EC and applicable local regulations.

## Discussion

We describe the protocol of the largest double-blind clinical trial to date on the balance of benefit and harm of adding low-dose GCs to the standard treatment of RA. It is a pragmatic trial positioned in the elderly where traditionally the evidence for treatment strategies is limited. Since the eligibility criteria of our trial are wide, we expect that many older patients with RA are eligible to participate in the study. Also, our design emulates the routine care setting. Assessments and procedures are tailored to represent standard of care, and concurrent antirheumatic treatment is allowed next to the study medication with minimal limitations.

The design also creates challenges. We have chosen harm as the co-primary outcome and tried to address potential confounding due to co-interventions, resulting in an elaborate decision tree to best interpret the results. Finally, we have tailored the cost analyses to align with the possible result of the benefit-harm assessment. The elderly population is also challenging to study, given the comorbidity, co-medication and frailty of these patients, increasing the chance of AEs and premature drop-out, and complicating the analysis.

We expect that the results of this trial are of importance for all rheumatologists who treat elderly patients with RA, and most likely will result in adjustment of the existing guidelines on RA treatment in elderly patients.

### Trial status

Recruitment started in June 2016 and will last until May 2018. This paper is based on protocol version 3.0 (18 May 2017).

## Additional files

Additional file 1: Protocol for sub-study of medication adherence. (DOCX 14 kb)

Additional file 2: SPIRIT Checklist. (DOC 121 kb)

Additional file 3: Full list of outcome measures: benefit, harm, and cost-utility. (DOCX 32 kb)

Additional file 4: List of ethical committees that approved the GLORIA trial. (DOCX 13 kb)

## Abbreviations

ACR: American College of Rheumatology
AE: Adverse event
CI: Confidence interval
DAS28: Disease Activity Score of 28 joints
DAS44: Disease Activity Score of 44 joints
DXA: Dual-energy X-ray absorptiometry
EULAR: European League Against Rheumatism
GC: Glucocorticoid
GCP: Good Clinical Practice
ICER: Incremental cost effectiveness ratio
ICH: International Council on Harmonisation of Technical Requirements for Registration of Pharmaceuticals for Human Use
IWRS: Interactive web response system
OtCM: Objective therapy Compliance monitoring technology
QoL: Quality of life
RA: Rheumatoid arthritis
RAID: Rheumatoid arthritis impact of disease
SAE: Serious adverse event
SUSAR: Suspected unexpected serious adverse drug reaction
TNF: tumour necrosis factor
