# Supplementary material for: Harm, benefit and costs associated with low-dose glucocorticoids added to the treatment strategies for rheumatoid arthritis in elderly patients (GLORIA trial): study protocol for a randomised controlled trial
Source: Trials. 2018 Jan 25;19:67. doi: 10.1186/s13063-017-2396-3 (PMC5785876; doi:10.1186/s13063-017-2396-3)
Supplement: Supplementary file 1 — Protocol for sub-study of medication adherence. (DOCX 14 kb) [file 13063_2017_2396_MOESM1_ESM.docx]

**Additional file 1**

**Appendix I: Protocol of substudy about medication adherence**

**Objective**

To study adherence, and test the effectiveness of smart device technology to improve adherence in a randomized substudy.

**In- and exclusion criteria**

All patients participating in the main trial are eligible to participate in the substudy, with the exception of patients who are not in the possession of a smart device or who prematurely discontinued the study medication within or at 3 months of the main trial.

**Randomization**

For the substudy to measure the effect of reminders to adherence, subjects with a smart device will be randomized to either receive or not receive an application loaded on their smart device that communicates with an adherence monitoring device in the cap of the drug bottle. In the substudy, randomization will be stratified for treatment and participating country (sample size of 144 patients is too small to stratify per center).

**Intervention**

For patients participating in the substudy, the electronic drug bottles will also be equipped with a wireless transmitter that can communicate real time with the smart device of the patient. Special software then can remind patients of the time of medication. The effectiveness of this application will be tested in the substudy: patients with a smart device will be randomized to receive or not receive the software (app) loaded on their smart device including subsequent reminders to take their medication for a period of 3 months. A reminder is triggered if a patient has not opened the bottle in 36 hours. If the patient does not open the bottle in the hour after the reminder, a second reminder will be send.

**Outcome measure**

Adherence to trial medication is measured through the e-communicative packaging solution as the count of days in which the bottle is opened on the appropriate days, as measured by the adherence cap. The cap is collected at every study visit and replaced by a new one. Each cap (identified by study ID only) is sent back to the provider and the data are read out centrally. At the end of the study, the adherence data is added to the study database.

**Analyses**

Analyses with the adherence data and pill count will be performed. The analysis plan for the medication adherence data will be developed separately.
