# Supplementary material for: Harm, benefit and costs associated with low-dose glucocorticoids added to the treatment strategies for rheumatoid arthritis in elderly patients (GLORIA trial): study protocol for a randomised controlled trial
Source: Trials. 2018 Jan 25;19:67. doi: 10.1186/s13063-017-2396-3 (PMC5785876; doi:10.1186/s13063-017-2396-3)
Supplement: Supplementary file 3 — Full list of outcome measures: benefit, harm, and cost-utility. (DOCX 32 kb) [file 13063_2017_2396_MOESM3_ESM.docx]

**Additional file 3**

**Appendix II: Full list of outcome measures: benefit, harm, and cost-utility**

*Analysis of benefit and harm in the first 3 months*

Primary outcomes will be analysed after 3 months of treatment like in the main analysis.

*Benefit: primary*

- Disease Activity Score of 28 joints (DAS28), index calculated from counts of 28 swollen and 28 tender joints, patient global assessment, and erythrocyte sedimentation rate (ESR) [17].

The DAS28 has a range of 0 to 9.4, in which levels ≥ 2.6 represents high disease activity.

- Radiographic damage of hands and forefeet, Sharp van der Heijde score [26]

*Benefit: secondary*

- WHO-ILAR core set of Rheumatoid Arthritis (RA) [21] outcome measures, comprising:
  - Pain (21-point Likert scale)
  - Patient and physician global assessment (21-point Likert scale)
  - Physical disability (Health Assessment Questionnaire)[27]
  - Joint counts (swollen joints and tender joints)

Joint swelling will be assessed as absent or present, joint tenderness will be graded semi-quantitatively from 0-3 according to the Disease Activity Score of 44 joints (DAS44) (Ritchie Articular Index) [28] and as absent or present for the Disease Activity Score of 28 joints (DAS28).

- - Acute phase reactants (C-reactive protein (CRP) and erythrocyte sedimentation rate (ESR))
  - Radiographs of hands and forefeet at 0 and 2 years (see primary outcome measures).
- DAS44: like the DAS28, but more joints and calculated differently [17].
- Severity and duration of morning stiffness

The patients are asked if they feel stiff during awakening in the morning. If the answer is ‘yes’, duration in minutes/hours is assessed and severity is assessed using a 21-point Likert Scale.

- The Short Form 36-item Health Survey (SF36) [29] and Euro-QoL in 5 dimensions (EQ-5D) [30]

Two questionnaires to assess quality of life.

- RA Impact of Disease (RAID) tool [31]

The RAID is a validated questionnaire assessing the 7 most important domains of impact of RA on patients, with a 21-point Likert Scale.

- (modified) Health Assessment Questionnaire (HAQ) [27]

The HAQ is a patient completed questionnaire specific for RA to assess physical functioning. The HAQ consists of 20 questions referring to 8 component sets: dressing/grooming, arising, eating, walking, hygiene, reach, grip, and activities. During the remote visits the modified HAQ is assessed, which consists of 10 questions.

*Harm: primary:*

- Occurrence of one or more serious adverse events (SAE) or an adverse event (AE) deemed of special interest

*Harm: secondary:*

Secondary outcomes regarding harm are:

- Vital signs, height, and weight. Vital signs include heart rate and blood pressure. Body weight and height will be obtained without shoes, jacket or coat.
- Bone mass assessed by Dual-energy X-ray Absorptiometry
- Discontinuation of study drug with reason. This includes patients in whom treatment with prednisolone becomes clinically indicated or those with unacceptable side effects attributable to study medication.
- Change of antirheumatic treatment, with reason
- Intensification of treatment for existing comorbidity, e.g. hypertension or diabetes
- Joint replacement surgery
- Patient symptom list. Patients will be asked to fill out the patient symptom list, which is a list with a number of physical complaints.
- Blood samples

Blood samples will be collected during the study as part of the standard of care of RA patients. The laboratory tests are described in detail in Table 2.

The time points of all measurements are described in Table 2.

*Economic evaluation*

An economic evaluation will be performed consisting of a cost-effectiveness and cost-utility analysis including health care costs, patient and family costs, and production losses, activity limitation, and work disability. Data will be obtained by a cost questionnaire completed three-monthly.
