# Supplementary material for: Harm, benefit and costs associated with low-dose glucocorticoids added to the treatment strategies for rheumatoid arthritis in elderly patients (GLORIA trial): study protocol for a randomised controlled trial
Source: Trials. 2018 Jan 25;19:67. doi: 10.1186/s13063-017-2396-3 (PMC5785876; doi:10.1186/s13063-017-2396-3)
Supplement: Supplementary file 4 — List of ethical committees that approved the GLORIA trial. (DOCX 13 kb) [file 13063_2017_2396_MOESM4_ESM.docx]

**Additional file 4**

**Appendix III: List of ethical committees that approved the GLORIA trial**

| **Country** | **Ethical committee** |
| --- | --- |
| Germany | Ethik-Kommission des Landes Berlin |
| Hungary | Medical research council Ethic Committee for clinical pharmacology |
| Italy | Regional Ethics Committee of Liguria |
| The Netherlands | VUmc METc |
| Portugal | CIEC |
| Romania | CNBMDM |
| Slovakia | Ethics Committee of National Institute of Rheumatic diseases – EC NÚRCH |
